# How socioeconomic status affected the access to health facilities and malaria diagnosis in children under five years: findings from 19 sub-Saharan African countries

**DOI:** 10.1186/s40249-023-01075-2

**Published:** 2023-04-06

**Authors:** Yue Ge, Di Liang, Jun Cao, Roland Gosling, Vivian Mushi, Jiayan Huang

**Affiliations:** 1 School of Public Health, Global Health Institute, Fudan University, Shanghai, China; 2 Key Laboratory of National Health Commission on Parasitic Disease Control and Prevention, Key Laboratory of Jiangsu Province on Parasite and Vector Control Technology, Jiangsu Institute of Parasitic Diseases, Wuxi, China; 3 Department of Disease Control, London School of Hygiene and Tropical Medicine, London, UK; 4 Department of Parasitology and Medical Entomology, Muhimbili University of Health and Allied Sciences, Dar es Salaam, Tanzania

**Keywords:** Malaria diagnosis, Children under five years of age, Socioeconomic status, Healthcare facility, Sub-Saharan Africa

## Abstract

**Background:**

Prompt and appropriate clinical management of malaria is critical for reducing the continued high burden of malaria among children under five years in sub-Saharan countries. However, more remains to be known about how a patient’s socioeconomic status (SES) would affect the access to diagnosis of malaria.

**Methods:**

In this cross-sectional study using the Demographic and Health Survey and Malaria Indicators Survey, we pooled the data of 38,567 febrile under-five children in 2016–2018 from 19 sub-Saharan countries. Multivariable logistic regression was used to assess the associations between SES and two binary outcomes: the visit to a health facility and a blood test for fever. Stratified analyses were further conducted by the type of health facilities (public hospitals/public primary healthcare facilities/private hospitals/private primary healthcare facilities) for the latter outcome.

**Results:**

Fifty-eight percent of the febrile children were taken to health facilities, among whom only 55% took blood tests. Compared to children from households in the highest wealth quintile, children in the lowest quintile were less likely to be taken to medical facilities [adjusted odds ratio (a*OR*) = 0.775, 95% confidence interval (*CI*): 0.675–0.889]. Parents with more than secondary education were more likely to seek care (a*OR* = 1.830, 95% *CI:* 1.561–2.145) and to have blood tests (a*OR* = 1.729, 95% *CI:*  1.436–2.082) for their febrile children than parents without formal education. The probabilities of receiving blood tests at public hospitals and public primary healthcare facilities stayed relatively high across parental education levels and wealth quintiles, while these probabilities remained the lowest at private primary healthcare facilities, ranging from 0.100 (95% *CI:* 0.074–0.127) to 0.139 (95% *CI:* 0.083–0.194) across parental education levels and from 0.104 (95% *CI:* 0.078–0.130) to 0.125 (95% *CI:* 0.090–0.160) across wealth quintiles.

**Conclusions:**

Significant socioeconomic disparities existed both in the access to health facilities and laboratory diagnosis of malaria in children in sub-Saharan African countries. These disparities were particularly evident in the private sector. Universal health coverage needs to be further strengthened to make formal healthcare in general and the laboratory diagnosis of malaria more accessible and affordable.

**Graphical abstract:**

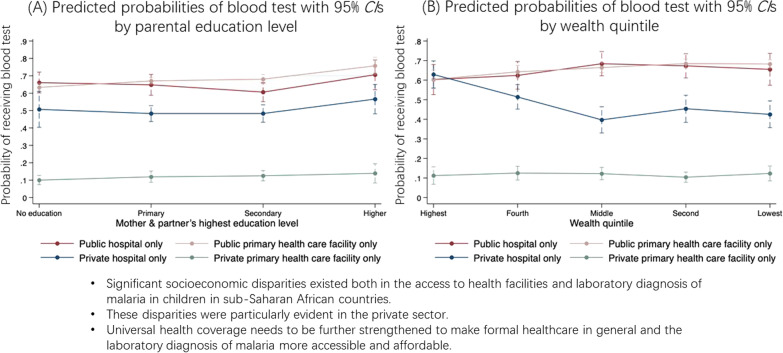

**Supplementary Information:**

The online version contains supplementary material available at 10.1186/s40249-023-01075-2.

## Background

In 2021, the World Health Organization (WHO) estimated that there were 247 million cases and 619,000 deaths of malaria in 84 malaria-endemic countries globally, and most of the malaria cases and deaths occurred in sub-Saharan Africa [1]. Children under 5 years bear a disproportionately high share of the global malaria burden, accounting for 67% of malaria deaths [2]. Children are at higher risk of contracting malaria and developing severe consequences, including death. Those children who survive often struggle with chronic anemia, seizures, or cognitive impairment, which severely hamper their growth, development, and attendance at school [3]. With evidence-based diagnostic and therapeutic procedures available, timely diagnosis and treatment among these children are vital to avoid the severe consequences above [4].

WHO recommends early diagnosis and prompt, effective treatment within 24–48 h of the onset of malaria symptoms (fever or a history of fever) [4]. Suspected malaria should be confirmed with microscopy or immunochromatographic rapid diagnostic tests (RDTs). The results of such a parasitological test should be available within a short time (< 2 h) of the patient presenting at the health facility [4]. Children with uncomplicated *Plasmodium falciparum* malaria should be treated with quality-assured artemisinin-based combination therapies (ACTs) [5, 6]. The algorithm for uncomplicated *Plasmodium falciparum* malaria diagnosis and treatment is demonstrated in Additional file 1: Fig. S1. However, current evidence suggests considerable gaps in the access to the laboratory diagnosis of malaria among children in sub-Saharan Africa, though WHO recommends a confirmatory blood test for all suspected cases of malaria.

First, febrile children often are not taken to health facilities for formal care [1, 7–12]. In 2015–2019, the mean treatment-seeking rate for febrile children under five years of age was 69% in sub-Saharan African countries, which only improved moderately from 65% in 2005–2011 [1]. The extent of this poor care-seeking behavior varies across countries. For instance, only 41% of febrile children from Ethiopia sought care in 2016, while 81% of febrile children from Liberia sought care in 2014 [13]. According to the current knowledge, care-seeking behavior can be affected by factors at the child level (e.g., age), factors at the caregiver level (e.g., education, perceived social norms regarding the treatment of fever), and factors at the household level [e.g., socioeconomic status (SES), type of residence (urban/rural), the travel time to health facilities [7, 9, 14]. Countries also vary in the distribution of related factors. For instance, it was estimated that the probabilities of care-seeking for fever at the nearest primary healthcare facility within 30-min travel time were 21% and 69% in Ethiopia and Uganda, respectively [7].

Second, blood tests for all suspected cases of malaria cannot be guaranteed even if formal care is sought, in which circumstances inappropriate treatment might occur thereafter [8, 15–21]. A cross-sectional study based on Malaria Indicators Surveys in 25 countries reported that 58% of febrile children under five years who had sought care received poor quality of case management for suspected malaria, with 62% receiving no blood tests, 82% receiving no antimalarial drug, and 72% receiving treatment more than 24 h after onset of fever [22]. This study suggested that regional disparities in malaria care quality could be driven by socioeconomic characteristics, but this study did not further examine these hypothesized associations. Another study using Service Provision Assessment (SPA) surveys also found that the majority of children treated for malaria across the nine surveyed sub-Saharan African countries did not receive a blood test diagnosis and an appropriate antimalarial [20]. But this study primarily focused on facility-level factors due to the lack of data on patient characteristics. Other single-country studies also explored the potential predictors of quality from the perspective of the health system, such as the availability of diagnostic tools, antimalarial medicines, and health workers [18, 19, 21, 23].

There is still much to know about the factors affecting the access to the laboratory diagnosis of malaria. Particularly, more remains to be known about how a patient’s SES would affect the access to the laboratory diagnosis of malaria. Lower SES has been widely recognized to be associated with higher malaria incidence and lower accessibility to malaria control interventions [10, 23, 24]. However, research on the association between SES and the receipt of blood tests once a patient has arrived at a health facility remains limited and inconclusive. Furthermore, it is unknown how facility-level factors might interact with a patient’s SES to influence the receipt of blood tests at a health facility. Lastly, most previous studies used national or sub-national data to examine the care-seeking and clinical management of malaria among children in sub-Saharan Africa [18–21]. Cross-country comparison is needed to understand how care-seeking behaviors and clinical practice vary across settings and possibly to find the common hurdles to the coverage of malaria diagnosis.

This study attempted to assess access to health facilities and the laboratory diagnosis of malaria among children. To fulfill the aim of the study, we examined the variation in the access to the laboratory diagnosis of malaria among children at the national and sub-national levels. Furthermore, we investigated how SES affected the access to the laboratory diagnosis of malaria among children, and whether such associations would be modified by the type of health facilities patients visited. The following hypotheses were formulated: febrile children with higher SES would be more likely to receive formal care; febrile children with higher SES would tend to receive blood tests once they arrived at a health facility, and this association would differ across the type of health facilities. The rationale for these hypotheses was that parents with higher SES might have greater awareness about the disease and more resources (e.g., transportation, cash) to seek healthcare.

## Methods

### Study design

We conducted a cross-sectional study in 19 malaria-endemic countries in sub-Sahara Africa (Benin, Burkina Faso, Burundi, Cameroon, Ghana, Guinea, Liberia, Madagascar, Malawi, Mali, Mozambique, Nigeria, Rwanda, Senegal, Sierra Leone, Tanzania, Togo, Uganda, and Zambia) using the Demographic Health Surveys (DHS) and the Malaria Indicators Surveys (MIS), both of which are repeated cross-sectional surveys (see detailed survey waves in Additional file 1: Table S1) [1]. We intended to include all DHS and MIS in a relatively tight time frame to facilitate cross-country comparison. We further restricted the study period to 2016–2018 to maximize the number of surveys included in this study.

### Data sources

DHS and MIS are both nationally representative surveys that provide data on a wide range of demographic and health topics, including malaria treatment. The sample of each survey is selected using a stratified two-stage cluster design. In the first stage of selection, the primary sampling units (PSUs) are selected from the list of census enumeration areas with probability proportional to the size within each stratum. In the second stage, a fixed number of households are selected by equal probability systematic sampling in each PSU. Trained staff visit the selected household and collect information on the characteristics of the household as well as women and men of reproductive age in the household. Women also answer questions about their children under the age of five, providing important information on childhood illness, mortality, vaccination, and nutrition. As a subset of the DHS survey, MIS collects data on ownership of mosquito nets and the prevention and treatment of malaria during high transmission seasons.

### Study population and sample size

To analyze the influence of maternal and family factors, we limited the sample to under-five children who lived with their mother and were usual residents of the family. We gathered data on 38,567 under-five children who reported being febrile in the last two weeks before the surveys. Among them, 22,180 children sought care. The unweighted samples for all treatment cascades are presented in Additional file 1: Fig. S2.

### Measurement of variables

#### Outcome measures

In malaria-endemic areas, malaria should be suspected in any patient presenting with a history of fever or temperature ≥ 37.5 ℃ with no other obvious cause, and suspected malaria should be confirmed with a parasitological test [4]. Accordingly, the first outcome indicated whether or not a febrile child was taken to a health facility which was defined as a public or private health facility except for a pharmacy. The second outcome indicated whether or not the child had blood tested for malaria parasites in any health facility. The first outcome was analyzed in all febrile children, while the second outcome was analyzed in children who sought care for the reported fever.

#### Explanatory variables

Based on the literature review and Anderson’s model of health service utilization, we included explanatory variables which might affect care-seeking behavior and the receipt of blood tests [25]. The following variables were included as they were available in both DHS and MIS: child’s sex, child’s age, mother’s age, mother and her partner’s highest education level, number of children in household, household wealth index, type of residence (urban/rural), type of health facilities to which the child was taken.

Among the explanatory variables mentioned above, the highest level of parental education and household wealth index were used to capture the child’s SES. Education level was classified into four categories: having no education, primary education (i.e., having attended primary school), secondary education (i.e., having attended secondary school), and higher education. Notably, as a composite measure of a household’s cumulative living standard, the wealth index was generated with principal components analysis using easy-to-collect data on a household’s ownership of selected assets, such as televisions and bicycles, materials used for housing construction, and types of water access and sanitation facilities. (For more detailed information, please see https://dhsprogram.com/Data/Guide-to-DHS-Statistics/index.cfm).

We categorized the types of health facilities into public hospitals, public primary health care (PHC) facilities, private hospitals, and private PHC facilities. Public hospitals indicated government hospitals. Public PHC facilities included government health centers, government health posts, mobile clinics, and community health workers that belonged to the public sector. Private hospitals indicated non-governmental hospitals. Private PHC facilities included private doctors, mobile clinics, and community health workers that belonged to the private sector.

### Statistical analysis

All statistical analyses were conducted among complete cases, using Stata version 15.1 (StataCorp, College Station, Texas, USA). Considering the small amount of missing data, bias due to missing data is unlikely to affect the results substantially (see Additional file 1: Fig. S2). All the descriptive analyses and regression below were weighted by survey weights provided by the DHS and MIS to account for the complex survey design.

First, we examined the percentage of febrile children who sought care and the percentage of care seekers who received blood tests. We assessed the variation in these proportions at the national and sub-national levels.

Second, we investigated the association between SES and care-seeking behavior as well as the receipt of blood tests. For care-seeking behavior, multivariable logistic regression was used, controlling for child’s sex, child’s age, mother’s age, mother and her partner’s highest education level, number of children in household, household wealth index, type of residence, and transmission season (dry/rainy). For the receipt of blood tests, the type of health facilities was further adjusted. Fewer than 3% of children who were taken to two or more facilities were excluded here, because DHS and MIS didn’t indicate in which facility these children received/didn’t receive blood tests. In all regression models, country fixed effects and year fixed effects were controlled for in all models to account for variations in malaria incidence across countries and years. We did not include the interaction term of education level and wealth index, which was not statistically significant (results not shown). Notably, variables only available in DHS or MIS were not included, such as the mother’s marriage, employment, and accessibility of health facilities. To assess the potential impact of omitting these variables, we controlled for these variables with only DHS data in the sensitivity analyses.

Furthermore, stratified analysis was conducted according to the type of health facilities for the receipt of blood tests, to further examine whether the associations between SES and receipt of blood tests differ across facility types. 0.61% of children (who were taken to only one facility) were taken to facilities that didn’t belong to the public sector or private sector (recorded as “other” in DHS and MIS). These records were excluded from the stratified analysis. Post-estimation simulations were conducted to generate the predicted probabilities of receiving blood tests at each type of facilities with other covariates at means as well as their confidence intervals.

## Results

### Characteristics of febrile children under five years and their households

We included 38,567 febrile children under five years from 19 malaria-endemic sub-Saharan African countries (see Additional file 1: Table S1). Of these children, 76% lived in rural areas, and 26% belonged to households in the lowest wealth quintile. There were 33% of children who had no parents with formal education (Table 1).

**Table 1:** Characteristics of febrile children under five and their households

Variables	Number	Percentage (%)
*Sex of child*		
Male	19,748	51.0
Female	18,948	49.0
*Age of child (months)*		
0–12	8988	23.2
13–24	9670	25.0
25–36	7655	19.8
37–48	6851	17.7
49–60	5532	14.3
*Age of mother (years)*		
15–19	2374	6.1
20–29	18,798	48.6
30–39	14,049	36.3
40–49	3474	9.0
*Mother and her partner’s highest level of education*		
No education	12,896	33.3
Primary	14,215	36.7
Secondary	9425	24.4
Higher	2159	5.6
*Wealth quintile*		
Lowest	10,031	25.9
Second	9151	23.6
Middle	7812	20.2
Fourth	6698	17.3
Highest	5002	12.9
*Number of children in the household*		
1	11,711	30.3
2	15,851	41.0
3 +	11,133	28.8
*Residence type*		
Rural	29,199	75.5
Urban	9497	24.5
*Season*		
Dry	27,556	71.2
Rainy	11,139	28.8
*Year*		
2016	8998	23.3
2017	11,086	28.7
2018	18,611	48.1
*Facility type*		
Public hospital	1965	9.1
Public PHC facility^a^	13,354	62.1
Private hospital	2331	10.8
Private PHC facility	3866	18.0

Data source: Demographic Health Surveys (DHS) and Malaria Indicators Surveys (MIS) in 2016–2018

Detailed country and wave information is presented in Additional file 1: Table S1. All estimates were calculated with survey weights (*n* = 38,695). Due to rounding, numbers may not total 38,695, and percentages may not total 100

*PHC* primary health care

^a^Sample: Febrile children under five who were taken to only one health facility except for the facility that was recorded as “other” in Demographic Health Surveys and Malaria Indicators Surveys (*n* = 21,517)

### National and sub-national variations in care-seeking behaviors and receipt of blood tests among febrile children under five years

**Fig. 1:**
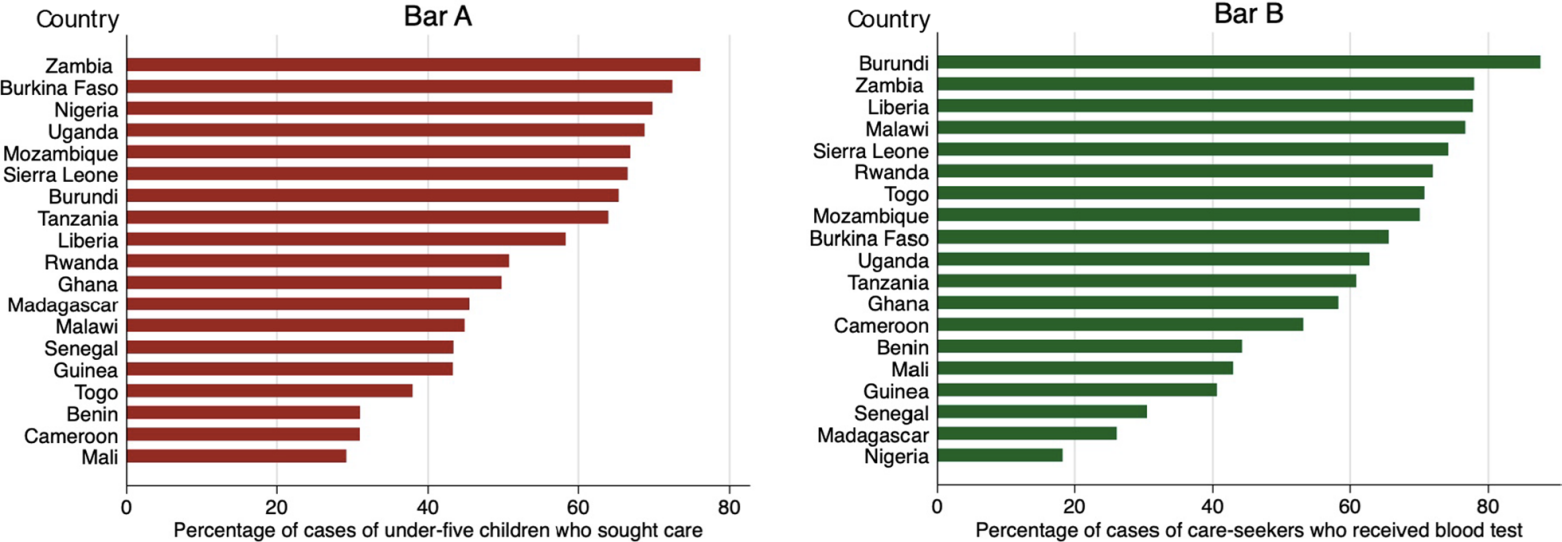
Percentage of febrile children under-five who sought care and percentage of care-seekers who received blood tests. **A** Percentage of under-five febrile children who sought care. **B** Percentage of care-seekers who received blood tests. All estimates were calculated using survey weights

On average, 58% of the febrile children under five years were taken to health facilities. Among those who were taken to health facilities, 71% were taken to the public sector. As shown in Fig. 1, there were wide variations in case-seeking behaviors across countries. The highest percentages of children who were taken to a health facility were in Zambia (76%, *n* = 1095), Burkina Faso (72%, *n* = 846), and Nigeria (70%, *n* = 4941). The lowest percentages of children who were taken to a health facility were in Mali (30%, *n* = 435), Cameroon (31%, *n* = 414), and Benin (31%, *n* = 741). Variations at the national level were also observed in the receipt of blood tests. Among the febrile children under five years who were taken to health facilities, 55% were reported to have taken a blood test. Sixty-four percent of the children in public facilities have taken a blood test, compared with only 32% in private facilities. Burundi had the highest percentage for blood tests at 88%, while Nigeria had the lowest record at 18%.

There were also extensive variations at the sub-national level. Tanzania showed the largest regional difference in care-seeking behaviors (53.9%) and the receipt of blood tests (98.7%) (see detailed regional differences in Additional file 1: Table S2).

### SES and the access to blood tests for malaria

As presented in Table 2, children from poorer households were less likely to be taken to health facilities. The odds of seeking care among febrile children from households in the second and lowest wealth quintile were only 0.850 (95% *CI*: 0.745–0.971) and 0.775 (95% *CI*: 0.675–0.889) times of the children from households in the highest quintile (see full regression results in Additional file 1: Table S3).

**Table 2:** Associations between socioeconomic status and care-seeking behaviors and receipt of blood tests for malaria in children

Variables	Care-seeking (*n* = 38,695)	Blood test (*n* = 21,513)^a^
	a*OR* 95% *CI*	a*OR* 95% *CI*
*Wealth quintile*		
Highest	1.000	1.000
Fourth	0.956 (0.847–1.080)	1.004 (0.847–1.190)
Middle	0.899 (0.791–1.022)	1.014 (0.853–1.205)
Second	0.850* (0.745–0.971)	1.076 (0.898–1.288)
Lowest	0.775*** (0.675–0.889)	1.091 (0.910–1.307)
*Mother & partner’s highest education level*		
No education	1.000	1.000
Primary	1.182*** (1.093–1.278)	1.109 (0.995–1.238)
Secondary	1.413*** (1.289–1.549)	1.142* (1.006–1.296)
Higher	1.830*** (1.561–2.145)	1.695*** (1.403–2.048)

Multiple logistic regressions were used. *OR*s were adjusted for sex, age, age of mother, type of residence, number of children in household, parents’ highest education level, country, survey year, and season. Survey weights were adjusted

*CI* Confidence interval, a*OR* adjusted odds ratio

*P < 0.05; ** *P* < 0.01; ***P < 0.001

^a^Sample: Febrile children under five who were taken to only one health facility except for the facility that was recorded as “other” in Demographic Health Surveys and Malaria Indicators Surveys

In the meantime, febrile children whose parents had more education had a better chance of seeking care and receiving blood tests (Table 2). Compared to parents with no formal education, the odds of seeking care for their febrile child were 1.182 times higher (95% *CI*: 1.093–1.278) among parents with primary education, 1.413 times higher (95% *CI*: 1.289–1.549) among parents with secondary education, and 1.830 times higher (95% *CI*: 1.561–2.145) among parents with higher education. Parents’ education level also showed a positive association with the likelihood of having blood tests. Compared to parents with no formal education, the odds of having blood tests were 1.142 times higher (95% *CI*: 1.006–1.296) among parents with secondary education, and 1.695 times higher (95% *CI*: 1.403–2.048) among parents with higher education (see full regression results in the online Additional file 1: Table S3).

For the results of sensitivity analyses, please see Additional file 1: Table S5.

### The modifying effect of the type of health facilities

As presented in Table 3, the type of health facilities modified the associations between socioeconomic status and receipt of blood tests. Among febrile children who visited public PHC facilities, compared to children from households in the highest wealth quintile, the odds of receiving blood tests were 1.294 times higher (95% *CI*: 1.030–1.626), 1.423 times higher (95% *CI*: 1.128–1.795), and 1.413 times higher (95% *CI*: 1.122–1.780) among children from households in the middle, second, and lowest quintiles, respectively. However, among febrile children who visited private hospitals, the odds of receiving blood tests among febrile children from households in the fourth, middle, second, and lowest wealth quintile were only 0.626 (95% *CI*: 0.427–0.917), 0.388 (95% *CI*: 0.252–0.599), 0.490 (95% *CI*: 0.313–0.769), and 0.437 (95% *CI*: 0.282–0.677) times of the children from households in the highest quintile (see full regression results in Additional file 1: Table S4).

**Table 3:** Associations between socioeconomic status and receipt of blood tests for malaria in children (stratified analysis)

Times	Public hospital		Public PHC		Private hospital		Private PHC	
	a*OR* 95% *CI*	*P* value	a*OR* 95% *CI*	*P* value	a*OR* 95% *CI*	*P* value	a*OR* 95% *CI*	*P* value
*Mother and partner’s highest education level*								
No education	1.000		1.000		1.000		1.000	
Primary	0.942 (0.647–1.371)	0.754	1.183** (1.044–1.340)	0.008	0.906 (0.578–1.418)	0.664	1.216 (0.830–1.780)	0.315
Secondary	0.786 (0.545–1.134)	0.198	1.238** (1.062–1.444)	0.006	0.908 (0.572–1.441)	0.681	1.286 (0.868–1.905)	0.210
Higher	1.227 (0.751–2.004)	0.415	1.814*** (1.366–2.409)	< 0.001	1.265 (0.734–2.181)	0.397	1.443 (0.820–2.540)	0.204
*Wealth quintile*								
Highest	1.000		1.000		1.000		1.000	
Fourth	1.092 (0.740–1.612)	0.658	1.180 (0.935–1.490)	0.162	0.626* (0.427–0.917)	0.016	1.126 (0.695–1.827)	0.629
Middle	1.424 (0.912–2.223)	0.120	1.294* (1.030–1.626)	0.027	0.388*** (0.252–0.599)	< 0.001	1.101 (0.661–1.833)	0.713
Second	1.353 (0.841–2.177)	0.212	1.423** (1.128–1.795)	0.003	0.490** (0.313–0.769)	0.002	0.919 (0.534–1.583)	0.762
Lowest	1.249 (0.728–2.145)	0.419	1.413** (1.122–1.780)	0.003	0.437*** (0.282–0.677)	0.000	1.108 (0.613–2.004)	0.733

Multiple logistic regressions were used. *OR*s were adjusted for sex, age, age of mother, type of residence, number of children in household, parents’ highest education level, country, survey year, and season. Survey weights were adjusted

Sample: Febrile children under five who were taken to only one health facility except for the facility that was recorded as “other” in Demographic Health Surveys and Malaria Indicators Surveys

*CI* confidence interval, a*OR* adjusted odds ratio

*P < 0.05; ** *P* < 0.01; ***P < 0.001

Meanwhile, higher levels of parents’ education indicated a better chance of receiving blood tests in public PHC facilities (Table 3). Children whose parents with primary, secondary, and higher education presented *OR*s of 1.183 (95% *CI*: 1.044–1.340), 1.238 (95% *CI*: 1.062–1.444), and 1.814 (95% *CI*: 1.366–2.409) to receive blood tests, respectively (see full regression results in Additional file 1: Table S4).

**Fig. 2:**
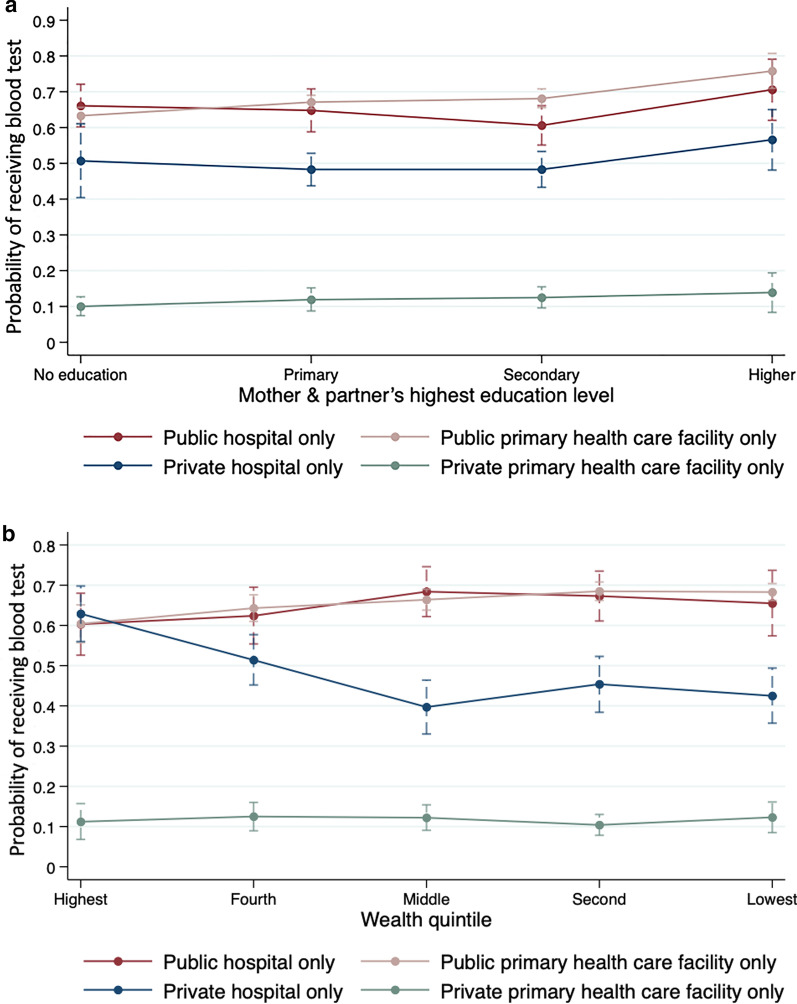
Predicted probability of receiving blood tests by SES across facility types. **A** Marginal mean probabilities and corresponding 95% *CI* of receiving blood tests by parental education level across facility types. **B** Marginal mean probabilities and corresponding 95% *CI* of receiving blood tests by wealth quintile across facility types. All estimates were calculated using survey weights. *CI* confidence interval

As presented in Fig. 2a, with other covariates at means, the predicted probabilities of receiving blood tests across facility types were highest at public PHC facilities for parents with at least some education, ranging from 0.671 (95% *CI*: 0.653–0.690) to 0.758 (95% *CI*: 0.710–0.807) by education level. These probabilities were lowest at private PHC facilities, ranging from 0.100 (95% *CI*: 0.074–0.127) to 0.139 (95% *CI*: 0.083–0.194) by education level.

As shown in Fig. 2b, with other covariates at means, the probabilities of receiving blood tests across facility types stayed high at both public PHC facilities and public hospitals across wealth quintiles, while these probabilities remained low at private PHC facilities, ranging from 0.104 (95% *CI*: 0.078–0.130) to 0.125 (95% *CI*: 0.090–0.160) by wealth quintile. Notably, the probabilities of receiving blood tests decreased from 0.629 (95% *CI*: 0.560–0.698) for children from the highest wealth quintile to 0.397 (95% *CI*: 0.330–0.464) for children from the middle wealth quintile, and these probabilities remained below 0.5 for the poorest two quintiles (see full predicted probabilities in Additional file 1: Table S6, S7).

## Discussion

Using recent DHS and MIS data, this study documented disparities in the access to health facilities and the laboratory diagnosis of malaria among children. Across the 19 sub-Sahara African countries included in this study, only 58% of the febrile children arrived at health facilities. Among those who ever visited a health facility, only 55% took a blood test. Significant disparities in the access to the laboratory diagnosis of malaria not only existed across countries and within a country but also manifested themselves on the SES ladder. Children of lower SES were disproportionately affected by the lack of access to quality malaria care. However, the influence of SES on the receipt of blood tests varied by where children sought care. Children from poorer households were more likely to receive blood tests in public PHC facilities, while less likely to receive blood tests in private hospitals.

Consistent with previous studies [8, 10, 26, 27], we found that febrile children with lower SES were less likely to be taken to a health facility. Such a relationship can be explained by Anderson’s model as part of predisposing (e.g., education, occupation, health beliefs) and enabling (e.g., financing factors, organizational factors) factors [25]. SES could influence healthcare utilization through various pathways. For instance, parents with better education might be more knowledgeable about malaria and seek care more promptly after symptom onset [11]. Wealthier families could have more financial resources for healthcare and other costs related to care-seeking (e.g., transportation) [10].

Furthermore, the impact of SES even persisted even after febrile children had arrived at health facilities, while there were only limited previous studies that drew mixed conclusions over this association. This study found that febrile children whose parents had higher levels of education were more likely to receive blood tests in general. This is possibly because better-educated parents had more knowledge about malaria and were more likely to report children’s symptoms accurately. It is also possible that parents with higher levels of education had more financial resources to pay for blood tests. But in a previous study using SPA data to assess the quality of malaria case management for children under five years, having a caregiver with primary or some secondary education was not significantly associated with the receipt of blood tests and recommended medications for malaria, after controlling for other patient-level, provider-level, and facility-level correlates [20]. In recent years (2013–2018), children with a caregiver having some secondary education were even significantly less likely to receive blood tests and recommended medications for malaria [20]. In another study in Mali, caregiver education was not found to be associated with incorrect case management of uncomplicated malaria among children under five years at public and private sector facilities [28]. The discrepancies in the associations between SES and quality of malaria case management might be explained by differences in research methods (e.g., different study populations, different sampling methods). In other fields of healthcare, the influence of patients’ SES on their clinical management has been documented around the world [29–31]. Low SES usually incur personal and financial strains for patients who do seek care [32].

However, the finding that the facility type modified how SES affected the quality of malaria case management suggested the potential protective role of the public sector in alleviating wealth disparities in malaria care. In public PHC facilities, febrile children from poorer families were more likely to receive blood tests. This might be a result of higher malaria prevalence among children from households with lower SES [33]. If this assumption held, among febrile children present at private hospitals, those who were from poorer households should also need blood tests more than their wealthier counterparts. In contrast, in private hospitals, children from wealthier families were more likely to receive blood tests. Thus, the wealth disparity could probably be explained by the differences in the ability to pay for blood tests. In public health facilities, blood tests might be more affordable to patients than those in the private sector, due to government subsidies. In other words, health facilities in the public sector might be more responsive to the health condition per se, while those in the private sector might be more sensitive to the ability to pay.

This study has the following strengths. First, this study added to the understanding of how SES influenced the coverage of malaria diagnosis. Access to the laboratory diagnosis of malaria among children required prompt care-seeking behavior on the parent side as well as the appropriate response on the provider side. Previous literature primarily focused on how SES affected parental behaviors, but few examined the role of SES once patients had arrived at health facilities. Second, this study used recent nationally representative data covering 19 sub-Saharan African countries. Therefore, the findings of this study could have important implications for this region.

This study has the following limitations. First, the associations reported in this study might not be interpreted as causal, as there might be uncontrolled confounders that were not available in both DHS and MIS data. For instance, variables on mother’s marriage, employment, and accessibility of health facilities were not available in MIS. In the sensitivity analysis, we controlled for these variables with only DHS data and generated similar results (Additional file 1: Table S5). Second, reverse causality could not be ruled out with the cross-sectional study design. Previous literature has discussed the potential reverse causality between the risk of malaria infection and SES [33]. In this study, there might be potential reverse causality between the lack of access to malaria care and households’ wealth index. Third, the information on malaria case management was self-reported by mothers, so there might be recall bias that can cause under or over-estimation of the response. Also, if a child was brought to two or more facilities, we cannot identify the facility which provided worse care. Fortunately, only 3% of children were taken to two or more facilities.

## Conclusions

In sub-Saharan African countries, febrile children with lower SES were less likely to have access to care, and even when they were brought to health facilities, they were less likely to receive a laboratory diagnosis of malaria. Health facilities in the public sector might alleviate the socioeconomic disparities in the access to malaria diagnosis to some extent. To narrow the socioeconomic disparities in the access to malaria diagnosis, universal health coverage needs to be further strengthened to make formal healthcare in general and the laboratory diagnosis of malaria more accessible and affordable. Furthermore, since a significant minority of febrile children sought care from the private sector, the affordability and quality of malaria care in the private sector warrant further improvement.

## Supplementary Information

**Additional file 1: Table S1.** Percentages of febrile children under five who sought care and percentages of care-seekers who received blood tests by country. **Table S2.** Regional differences in care-seeking behaviors and receipt of blood tests by country. **Table S3.** Regression results on determinants of care-seeking behaviors and receipt of blood tests for malaria in children. **Table S4.** Regression results on determinants of blood tests for malaria in children (Stratified analysis). **Table S5.** Regression results on determinants of care-seeking behaviors and receipt of blood tests for malaria in children (sensitive analyses). **Table S6.** Predicted probability of receiving blood tests with 95% *CI*s by mother & partner’s highest education level across facility types. **Table S7.** Predicted probability of receiving blood tests with 95% *CI*s by wealth quintile across facility types. **Fig. S1.** Algorithm for uncomplicated *Plasmodium falciparum* malaria diagnosis and treatment. Note: This figure was generated by the authors according to the WHO 2021 Guidelines for malaria. a Danger signs for severe falciparum malaria can be found in WHO 2021 Guidelines for malaria^[5]^.b For the treatment after admission, please see World Health Organization, Universal access to malaria diagnostic testing – An operational manual, 2013.c Pre-referral treatment as recommended by WHO 2021 Guidelines for malaria^[5]^. **Fig. S2.** Flow diagram of samples for each treatment cascade. Note: All numbers are unweighted. **Fig. S3.** Crosstabs of facility type and children’s SES. Distribution of facility type by the highest education level of children’s parents. (B) Distribution of facility type by wealth quintile. (C) Distribution of the highest education level of children’s parents by facility type. (D) Distribution of wealth quintile by facility type.

## Data Availability

The datasets analyzed during the current study are available in the DHS Program, https://dhsprogram.com/data/available-datasets.cfm. Not applicable.

## Abbreviations

SES: Socioeconomic status
*CI*: Confidence interval
WHO: World Health Organization
RDTs: Rapid diagnostic tests
ACTs: Artemisinin-based combination therapies
SPA: Service Provision Assessment
DHS: Demographic Health Surveys
MIS: Malaria Indicators Surveys
PSUs: Primary sampling units
PHC: Primary healthcare
