# Supplementary material for: How socioeconomic status affected the access to health facilities and malaria diagnosis in children under five years: findings from 19 sub-Saharan African countries
Source: Infect Dis Poverty. 2023 Apr 6;12:29. doi: 10.1186/s40249-023-01075-2 (PMC10077698; doi:10.1186/s40249-023-01075-2)

**Table S1. Percentages of febrile children under five who sought care and percentages of care-seekers who received blood tests by country**

| **Country** | **Survey** | **Year** | ***N*** | **Sought care** | | **Blood tested** | |
| --- | --- | --- | --- | --- | --- | --- | --- |
|  |  |  |  | ***n*** | **%** | ***n*** | **%** |
| Benin | DHS | 2018 | 2391 | 741 | 31 | 328 | 44.3 |
| Burkina Faso | MIS | 2018 | 1168 | 846 | 72.4 | 553 | 65.6 |
| Burundi | DHS | 2017 | 5001 | 3,266 | 65.3 | 2,861 | 87.6 |
| Cameroon | DHS | 2018 | 1338 | 414 | 31 | 220 | 53.2 |
| Ghana | MIS | 2016 | 909 | 452 | 49.7 | 263 | 58.3 |
| Guinea | DHS | 2018 | 1170 | 506 | 43.3 | 205 | 40.6 |
| Liberia | MIS | 2016 | 994 | 580 | 58.3 | 451 | 77.7 |
| Madagascar | MIS | 2016 | 1082 | 492 | 45.5 | 128 | 26.1 |
| Malawi | MIS | 2017 | 1024 | 459 | 44.8 | 352 | 76.6 |
| Mali | DHS | 2018 | 1491 | 435 | 29.2 | 187 | 42.9 |
| Mozambique | MIS | 2018 | 1441 | 963 | 66.9 | 675 | 70 |
| Nigeria | DHS | 2018 | 7081 | 4,941 | 69.8 | 901 | 18.2 |
| Rwanda | MIS | 2017 | 876 | 444 | 50.7 | 320 | 71.9 |
| Senegal | DHS | 2017, 2018 | 3156 | 1,368 | 43.3 | 418 | 30.5 |
| Sierra Leone | MIS | 2016 | 1523 | 1,013 | 66.5 | 751 | 74.2 |
| Tanzania | MIS | 2017 | 1392 | 890 | 64 | 542 | 60.9 |
| Togo | MIS | 2017 | 731 | 277 | 37.9 | 196 | 70.7 |
| Uganda | DHS | 2016 | 4490 | 3,086 | 68.7 | 1,938 | 62.8 |
| Zambia | DHS | 2018 | 1438 | 1,095 | 76.1 | 853 | 77.9 |
| Total | - | - | 38,695 | 22,269 | 57.6 | 12,142 | 54.5 |

Data source: Demographic Health Survey and Malaria Indicators Survey in 2016-2018. All estimates were calculated using survey weight.

**Table S2. Regional differences in care-seeking behaviors and receipt of blood tests by country**

|  | **Sought care** | | | **Blood tested** | | | |
| --- | --- | --- | --- | --- | --- | --- | --- |
| **Country** | **Min. %** | **Max. %** | **Diff.** | | **Min. %** | **Max. %** | **Diff.** |
| Benin | 23.2 | 50.4 | 27.2 | | 29.1 | 70.7 | 41.6 |
| Burkina Faso | 52.1 | 85.9 | 33.8 | | 54.0 | 90.1 | 36.1 |
| Burundi | 41.9 | 78.2 | 36.3 | | 43.2 | 97.9 | 54.7 |
| Cameroon | 19.5 | 52.1 | 32.6 | | 31.7 | 82.9 | 51.2 |
| Ghana | 37.5 | 84.4 | 46.9 | | 44.7 | 80.0 | 35.3 |
| Guinea | 21.5 | 58.8 | 37.3 | | 23.4 | 53.8 | 30.4 |
| Liberia | 54.3 | 69.5 | 15.2 | | 68.0 | 91.2 | 23.2 |
| Madagascar | 27.2 | 75.8 | 48.6 | | 3.5 | 69.1 | 65.6 |
| Malawi | 36.2 | 60.9 | 24.7 | | 74.8 | 78.1 | 3.3 |
| Mali | 9.5 | 44.2 | 34.7 | | 11.1 | 56.6 | 45.5 |
| Mozambique | 49.6 | 83.1 | 33.5 | | 44.5 | 80.2 | 35.7 |
| Nigeria | 55.8 | 79.8 | 24.0 | | 13.8 | 36.4 | 22.6 |
| Rwanda | 40.1 | 55.4 | 15.3 | | 51.1 | 84.8 | 33.7 |
| Senegal | 27.3 | 56.1 | 28.8 | | 7.6 | 43.2 | 35.6 |
| Sierra Leone | 51.0 | 70.2 | 19.2 | | 53.6 | 82.9 | 29.3 |
| Tanzania | 46.1 | 100.0 | 53.9 | | 0.0 | 98.7 | 98.7 |
| Togo | 28.0 | 58.8 | 30.8 | | 54.8 | 88.3 | 33.5 |
| Uganda | 49.4 | 88.5 | 39.1 | | 40.4 | 77.7 | 37.3 |
| Zambia | 65.8 | 84.6 | 18.8 | | 27.1 | 92.1 | 65.0 |

Data source: Demographic Health Survey and Malaria Indicators Survey in 2016–2018. All estimates were calculated using survey weights

**Table S3. Regression results on determinants of care-seeking behaviors and receipt of blood tests for malaria in children**

| **Variables** | **Care-seeking** | | **Blood tests** † | |
| --- | --- | --- | --- | --- |
|  | **a*OR*** | **95% *CI*** | **a*OR*** | **95% *CI*** |
| **Mother & partner’s highest education level** |  |  |  |  |
| No education | 1.000 |  | 1.000 |  |
| Primary | 1.182*** | 1.093–1.278 | 1.109 | 0.995–1.238 |
| Secondary | 1.413*** | 1.289–1.549 | 1.142* | 1.006–1.296 |
| Higher | 1.830*** | 1.561–2.145 | 1.695*** | 1.403–2.048 |
| **Wealth quintile** |  |  |  |  |
| Highest | 1.000 |  | 1.000 |  |
| Fourth | 0.956 | 0.847–1.080 | 1.004 | 0.847–1.190 |
| Middle | 0.899 | 0.791–1.022 | 1.014 | 0.853–1.205 |
| Second | 0.850* | 0.745–0.971 | 1.076 | 0.898–1.288 |
| Lowest | 0.775*** | 0.675–0.889 | 1.091 | 0.910–1.307 |
| **Child’s sex** |  |  |  |  |
| Female | 1.000 |  | 1.000 |  |
| Male | 1.023 | 0.973–1.076 | 0.999 | 0.926–1.078 |
| **Child’s age (months)** |  |  |  |  |
| 0–12 | 1.000 |  | 1.000 |  |
| 13–24 | 1.003 | 0.933–1.079 | 1.538*** | 1.382–1.713 |
| 25–36 | 0.881** | 0.817–0.951 | 1.699*** | 1.521–1.898 |
| 37–48 | 0.842*** | 0.778–0.910 | 1.682*** | 1.487–1.903 |
| 49–60 | 0.774*** | 0.711–0.841 | 1.833*** | 1.612–2.085 |
| **Mother’s age (years)** |  |  |  |  |
| 15–19 | 1.000 |  | 1.000 |  |
| 20–29 | 1.103 | 0.982–1.240 | 1.122 | 0.956–1.318 |
| 30–39 | 1.133* | 1.003–1.278 | 1.153 | 0.974–1.364 |
| 40–49 | 1.084 | 0.939–1.251 | 1.076 | 0.875–1.324 |
| **Type of residence** |  |  |  |  |
| Rural | 1.000 |  | 1.000 |  |
| Urban | 0.999 | 0.904–1.103 | 0.982 | 0.861–1.120 |
| **Number of children in the household** |  |  |  |  |
| 1 | 1.000 |  | 1.000 |  |
| 2 | 0.927* | 0.870–0.988 | 0.984 | 0.894–1.083 |
| 3+ | 0.877*** | 0.814–0.945 | 0.910 | 0.816–1.014 |
| **Facility type** |  |  |  |  |
| Public hospital | - | - | 1.000 |  |
| Public PHC facility |  |  | 0.765** | 0.651–0.900 |
| Private hospital |  |  | 0.385*** | 0.316–0.469 |
| Private PHC facility | - | - | 0.237*** | 0.195–0.287 |
| **Season** |  |  |  |  |
| Dry | 1.000 |  | 1.000 |  |
| Rainy | 1.333 | 0.956 - 1.858 | 2.449*** | 1.537 - 3.902 |
| **Survey year** |  |  |  |  |
| 2016 | 1.000 |  | 1.000 |  |
| 2017 | 0.218*** | 0.165–0.287 | 0.490*** | 0.330–0.726 |
| 2018 | 0.213*** | 0.179–0.253 | 0.428*** | 0.346–0.530 |
| **Constant** | 1.637*** | 1.362–1.967 | 1.633** | 1.191–2.241 |
| **N** | 38,695 |  | 21,513 |  |

Note: Multiple logistic regression was used with country fixed effects (not shown). We reported adjusted odds ratios (a*OR*) and corresponding 95% *CI*, which have been adjusted for survey weights. **P* < 0.05; ** *P* < 0.01; ****P* < 0.001.

†Sample: Febrile children under five who were taken to only one medical facility except the facility that was recorded as “other” in DHS and MIS.

**Table S4. Regression results on determinants of blood tests for malaria in children (Stratified analysis)**

| **Variables** | **Public hospital** | | | **Public PHC** | | | **Private hospital** | | | **Private PHC** | | |
| --- | --- | --- | --- | --- | --- | --- | --- | --- | --- | --- | --- | --- |
|  | **a*OR*** | **95% *CI*** | ***P* value** | **a*OR*** | **95% *CI*** | ***P* value** | **a*OR*** | **95% *CI*** | ***P* value** | **a*OR*** | **95% *CI*** | ***P* value** |
| **Mother & partner’s highest education level** |  |  |  |  |  |  |  |  |  |  |  |  |
| No education | 1.000 |  |  | 1.000 |  |  | 1.000 |  |  | 1.000 |  |  |
| Primary | 0.942 | 0.647–1.371 | 0.754 | 1.183** | 1.044–1.340 | 0.008 | 0.906 | 0.578–1.418 | 0.664 | 1.216 | 0.830–1.780 | 0.315 |
| Secondary | 0.786 | 0.545–1.134 | 0.198 | 1.238** | 1.062–1.444 | 0.006 | 0.908 | 0.572–1.441 | 0.681 | 1.286 | 0.868–1.905 | 0.210 |
| Higher | 1.227 | 0.751–2.004 | 0.415 | 1.814*** | 1.366–2.409 | 0.000 | 1.265 | 0.734–2.181 | 0.397 | 1.443 | 0.820–2.540 | 0.204 |
| **Wealth quintile** |  |  |  |  |  |  |  |  |  |  |  |  |
| Highest | 1.000 |  |  | 1.000 |  |  | 1.000 |  |  | 1.000 |  |  |
| Fourth | 1.092 | 0.740–1.612 | 0.658 | 1.180 | 0.935–1.490 | 0.162 | 0.626* | 0.427–0.917 | 0.016 | 1.126 | 0.695–1.827 | 0.629 |
| Middle | 1.424 | 0.912–2.223 | 0.120 | 1.294* | 1.030–1.626 | 0.027 | 0.388*** | 0.252–0.599 | 0.000 | 1.101 | 0.661–1.833 | 0.713 |
| Second | 1.353 | 0.841–2.177 | 0.212 | 1.423** | 1.128–1.795 | 0.003 | 0.490** | 0.313–0.769 | 0.002 | 0.919 | 0.534–1.583 | 0.762 |
| Lowest | 1.249 | 0.728–2.145 | 0.419 | 1.413** | 1.122–1.780 | 0.003 | 0.437*** | 0.282–0.677 | 0.000 | 1.108 | 0.613–2.004 | 0.733 |
| **Child’s sex** |  |  |  |  |  |  |  |  |  |  |  |  |
| Female | 1.000 |  |  | 1.000 |  |  | 1.000 |  |  | 1.000 |  |  |
| Male | 0.968 | 0.757–1.238 | 0.797 | 0.990 | 0.901–1.088 | 0.831 | 0.933 | 0.764–1.139 | 0.495 | 1.056 | 0.819–1.362 | 0.672 |
| **Child’s age(month)** |  |  |  |  |  |  |  |  |  |  |  |  |
| 0–12 | 1.000 |  |  | 1.000 |  |  | 1.000 |  |  | 1.000 |  |  |
| 13–24 | 2.047*** | 1.423–2.946 | 0.000 | 1.570*** | 1.382–1.784 | 0.000 | 1.084 | 0.788–1.491 | 0.619 | 1.534* | 1.036–2.270 | 0.033 |
| 25–36 | 2.365*** | 1.607–3.481 | 0.000 | 1.757*** | 1.535–2.012 | 0.000 | 1.291 | 0.941–1.771 | 0.113 | 1.484 | 0.993–2.217 | 0.054 |
| 37–48 | 2.043*** | 1.389–3.005 | 0.000 | 1.769*** | 1.523–2.054 | 0.000 | 1.056 | 0.745–1.497 | 0.758 | 1.674* | 1.081–2.591 | 0.021 |
| 49–60 | 1.938** | 1.293–2.903 | 0.001 | 1.978*** | 1.688–2.317 | 0.000 | 1.309 | 0.918–1.867 | 0.136 | 1.681* | 1.068–2.645 | 0.025 |
| **Mother’s age** |  |  |  |  |  |  |  |  |  |  |  |  |
| 15–19 | 1.000 |  |  | 1.000 |  |  | 1.000 |  |  | 1.000 |  |  |
| 20–29 | 1.177 | 0.703–1.971 | 0.534 | 1.056 | 0.876–1.273 | 0.567 | 1.251 | 0.783–2.000 | 0.349 | 2.093* | 1.092–4.011 | 0.026 |
| 30–39 | 1.250 | 0.734–2.130 | 0.411 | 1.121 | 0.920–1.365 | 0.259 | 1.172 | 0.715–1.920 | 0.528 | 2.071* | 1.048–4.091 | 0.036 |
| 40–49 | 1.057 | 0.537–2.081 | 0.872 | 1.034 | 0.810–1.322 | 0.787 | 1.498 | 0.824–2.724 | 0.185 | 1.454 | 0.640–3.302 | 0.371 |
| **Type of residence** |  |  |  |  |  |  |  |  |  |  |  |  |
| Rural | 1.000 |  |  | 1.000 |  |  | 1.000 |  |  | 1.000 |  |  |
| Urban | 0.872 | 0.610–1.246 | 0.452 | 0.946 | 0.797–1.122 | 0.522 | 0.865 | 0.623–1.201 | 0.386 | 1.403 | 0.970–2.031 | 0.072 |
| **Number of children** |  |  |  |  |  |  |  |  |  |  |  |  |
| 1 | 1.000 |  |  | 1.000 |  |  | 1.000 |  |  | 1.000 |  |  |
| 2 | 0.954 | 0.703–1.295 | 0.762 | 1.051 | 0.932–1.185 | 0.421 | 0.715** | 0.560–0.913 | 0.007 | 1.181 | 0.858–1.627 | 0.308 |
| 3+ | 0.864 | 0.623–1.198 | 0.381 | 0.955 | 0.833–1.094 | 0.507 | 0.654** | 0.474–0.903 | 0.010 | 1.159 | 0.832–1.614 | 0.382 |
| **Season** |  |  |  |  |  |  |  |  |  |  |  |  |
| Dry | 1.000 |  |  | 1.000 |  |  | 1.000 |  |  | 1.000 |  |  |
| Rainy | 0.954 | 0.703–1.295 | 0.032 | 2.305** | 1.343–3.955 | 0.002 | 1.979 | 0.292–13.412 | 0.484 | 1.236 | 0.069–22.192 | 0.886 |
| **Survey year** |  |  |  |  |  |  |  |  |  |  |  |  |
| 2016 | 1.000 |  |  | 1.000 |  |  | 1.000 |  |  | 1.000 |  |  |
| 2017 | 0.075** | 0.014–0.405 | 0.003 | 0.437*** | 0.283–0.675 | 0.000 | 0.389 | 0.063–2.402 | 0.309 | 0.313 | 0.028–3.504 | 0.346 |
| 2018 | 0.085*** | 0.040–0.179 | 0.000 | 0.385*** | 0.292–0.506 | 0.000 | 0.441*** | 0.281–0.691 | 0.000 | 0.185*** | 0.077–0.446 | 0.000 |
| **Constant** | 4.797*** | 1.944–11.837 | 0.001 | 1.207 | 0.845–1.724 | 0.300 | 1.742 | 0.782–3.879 | 0.174 | 0.286* | 0.103–0.794 | 0.016 |
| **N** | 1959 |  |  | 13352 |  |  | 2329 |  |  | 3858 |  |  |

Note: Multiple logistic regressions were used. Survey weights were also adjusted. **P* < 0.05; ** *P* < 0.01; ****P* < 0.001.

Sample: Febrile children under five who were taken to only one medical facility except the facility that was recorded as “other” in DHS and MIS.

**Table S5. Regression results on determinants of care-seeking behaviors and receipt of blood tests for malaria in children (sensitive analyses)**

| **Variable** | **Care-seeking** | | **Blood tests** | |
| --- | --- | --- | --- | --- |
|  | **a*OR*** | **95% *CI*** | **a*OR*** | **95% *CI*** |
| **Mother & partner’s highest education level** |  |  |  |  |
| No education | 1.000 |  | 1.000 |  |
| Primary | 1.215*** | 1.110–1.331 | 1.163* | 1.015–1.332 |
| Secondary | 1.436*** | 1.294–1.593 | 1.177* | 1.014–1.366 |
| Higher | 1.745*** | 1.484–2.052 | 1.687*** | 1.373–2.073 |
| **Wealth quintile** |  |  |  |  |
| Highest | 1.000 |  | 1.000 |  |
| Fourth | 0.944 | 0.817–1.091 | 0.932 | 0.756–1.150 |
| Middle | 0.882 | 0.759–1.025 | 0.960 | 0.783–1.176 |
| Second | 0.867 | 0.743–1.012 | 1.087 | 0.877–1.348 |
| Lowest | 0.820* | 0.698–0.963 | 1.071 | 0.863–1.329 |
| **Child’s sex** |  |  |  |  |
| Female | 1.000 |  | 1.000 |  |
| Male | 1.070* | 1.009–1.135 | 0.992 | 0.910–1.080 |
| **Child’s age(month)** |  |  |  |  |
| 0–12 | 1.000 |  | 1.000 |  |
| 13–24 | 1.075 | 0.987–1.170 | 1.503*** | 1.315–1.719 |
| 25–36 | 0.980 | 0.896–1.071 | 1.665*** | 1.456–1.903 |
| 37–48 | 1.008 | 0.920–1.104 | 1.572*** | 1.355–1.824 |
| 49–60 | 0.899* | 0.815–0.993 | 1.820*** | 1.563–2.119 |
| **Mother’s age** |  |  |  |  |
| 15–19 | 1.000 |  | 1.000 |  |
| 20–29 | 1.144 | 0.997–1.312 | 1.060 | 0.876–1.282 |
| 30–39 | 1.124 | 0.970–1.302 | 1.045 | 0.858–1.272 |
| 40–49 | 1.113 | 0.940–1.317 | 1.016 | 0.796–1.298 |
| **Mother lived with partner** |  |  |  |  |
| No | 1.000 |  | 1.000 |  |
| Yes | 1.004 | 0.900–1.120 | 0.941 | 0.797–1.111 |
| **Mother got employed** |  |  |  |  |
| No | 1.000 |  | 1.000 |  |
| Yes | 1.192*** | 1.098–1.293 | 0.923 | 0.825–1.033 |
| **Type of residence** |  |  |  |  |
| Rural | 1.000 |  | 1.000 |  |
| Urban | 1.021 | 0.911–1.145 | 0.991 | 0.847–1.160 |
| **Number of children** |  |  |  |  |
| 1 | 1.000 |  | 1.000 |  |
| 2 | 0.959 | 0.890–1.035 | 0.991 | 0.886–1.109 |
| 3+ | 0.927 | 0.851–1.009 | 0.888 | 0.783–1.007 |
| **Access to health facility is a big problem** |  |  |  |  |
| No | 1.000 |  | 1.000 |  |
| Yes | 0.720*** | 0.669–0.776 | 0.965 | 0.866–1.076 |
| **Facility type** |  |  |  |  |
| Public hospital | - | - | 1.000 | - |
| Public PHC facility |  |  | 0.674*** | 0.547–0.829 |
| Private hospital |  |  | 0.278*** | 0.217–0.357 |
| Private PHC facility | - | - | 0.184*** | 0.146–0.232 |
| **Season** |  |  |  |  |
| Dry | - | - | - | - |
| Rainy | - | - | - | - |
| **Survey year** |  |  |  |  |
| 2016 | 1.000 |  | 1.000 |  |
| 2017 | 0.212*** | 0.161–0.280 | 0.472*** | 0.316–0.706 |
| 2018 | 0.207*** | 0.174–0.246 | 0.408*** | 0.327–0.510 |
| **Constant** | 1.435** | 1.151–1.789 | 2.557*** | 1.723–3.795 |
| ***n*** | 27,556 |  | 15,447 |  |

Note: Multiple logistic regressions were used. Survey weights were also adjusted. **P* < 0.05; ** *P* < 0.01; ****P* < 0.001.

**Table S6. Predicted probability of receiving blood tests with 95% *CI*s by mother & partner’s highest education level across facility types**

| **Facility type** | **Mother & partner’s highest education level** | | | |
| --- | --- | --- | --- | --- |
|  | **No education** | **Primary** | **Secondary** | **Higher** |
| Public hospital | 0.661  (0.602, 0.721) | 0.648  (0.588, 0.708) | 0.606  (0.551, 0.661) | 0.706  (0.620, 0.791) |
| Public PHC facility | 0.633  (0.611, 0.656) | 0.671  (0.653, 0.690) | 0.681  (0.655, 0.708) | 0.758  (0.710, 0.807) |
| Private hospital | 0.507  (0.404, 0.610) | 0.483  (0.437, 0.528) | 0.483  (0.433, 0.533) | 0.566  (0.481, 0.650) |
| Private PHC facility | 0.100  (0.074, 0.127) | 0.119  (0.087, 0.152) | 0.125  (0.096, 0.155) | 0.139  (0.083, 0.194) |

**Table S7. Predicted probability of receiving blood tests with 95% *CI*s by wealth quintile across facility types**

| **Facility type** | **Wealth quintile** | | | | |
| --- | --- | --- | --- | --- | --- |
|  | **Highest** | **Fourth** | **Middle** | **Second** | **Lowest** |
| Public hospital | 0.603  (0.526, 0.680) | 0.624  (0.554, 0.695) | 0.684  (0.622, 0.746) | 0.673  (0.611, 0.735) | 0.655  (0.574, 0.737) |
| Public PHC facility | 0.604  (0.557, 0.651) | 0.643  (0.610, 0.676) | 0.664  (0.638, 0.690) | 0.685  (0.662, 0.708) | 0.683  (0.662, 0.704) |
| Private hospital | 0.629  (0.560, 0.698) | 0.514  (0.452, 0.577) | 0.397  (0.330, 0.464) | 0.454  (0.384, 0.523) | 0.425  (0.357, 0.494) |
| Private PHC facility | 0.112  (0.068, 0.157) | 0.125  (0.090, 0.160) | 0.122  (0.091, 0.154) | 0.104  (0.078, 0.130) | 0.123  (0.085, 0.161) |

**
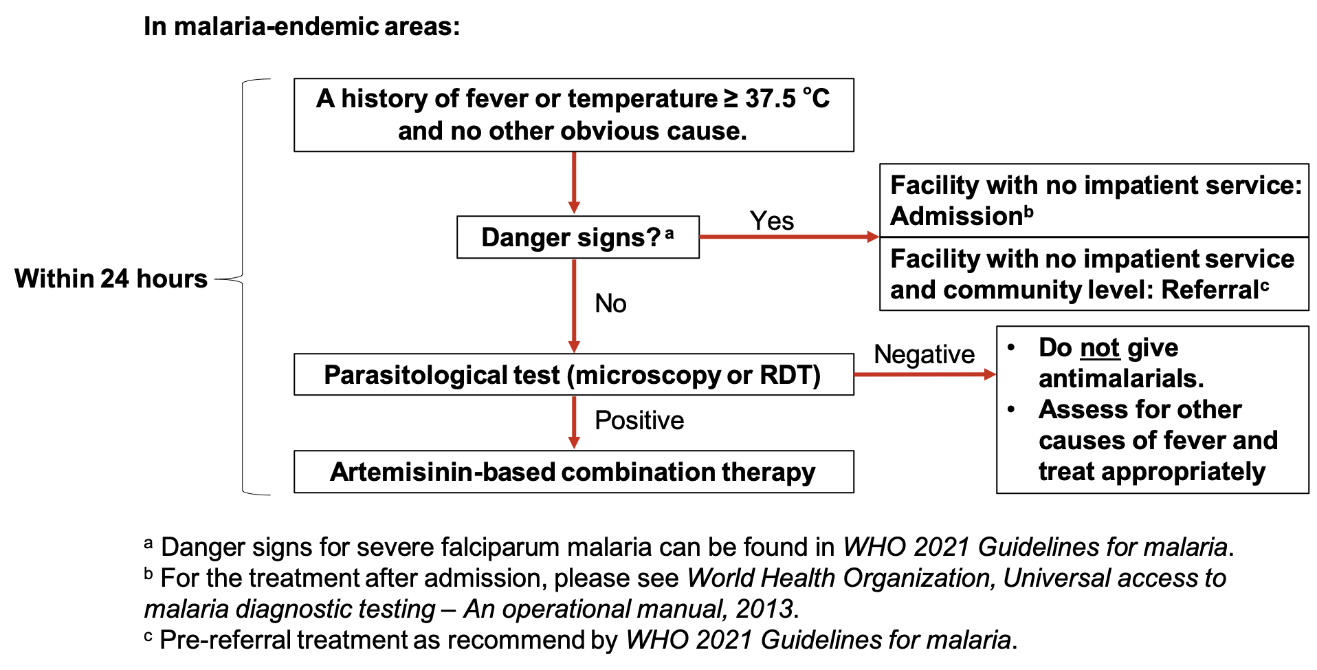
Figure S1. Algorithm for uncomplicated *Plasmodium falciparum* malaria diagnosis and treatment**

Note: This figure was generated by the authors according to the WHO 2021 Guidelines for malaria.

a Danger signs for severe falciparum malaria can be found in WHO 2021 Guidelines for malaria^5^.

b For the treatment after admission, please see World Health Organization, Universal access to malaria diagnostic testing – An operational manual, 2013.^43^

c Pre-referral treatment as recommended by WHO 2021 Guidelines for malaria^5^.

**Figure S2. Flow diagram of samples for each treatment cascade**


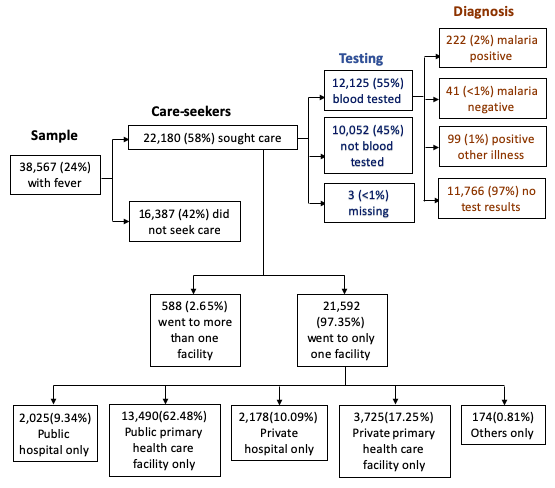


Note: All numbers are unweighted.

**Figure S3. Crosstabs of facility type and children’s SES**

1. Distribution of facility type by the highest education level of children’s parents


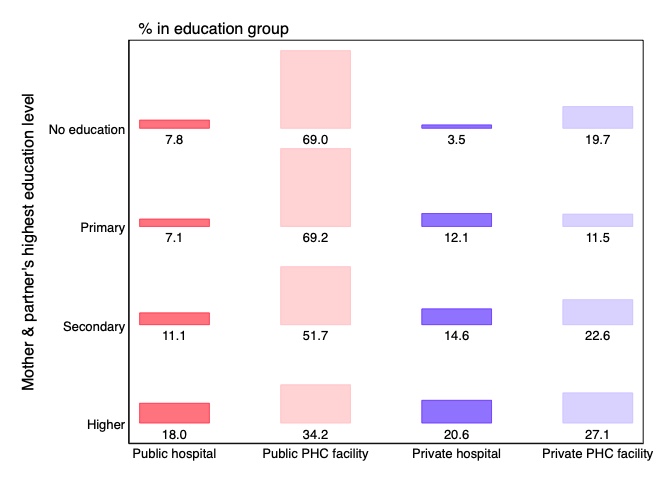


(B) Distribution of facility type by wealth quintile


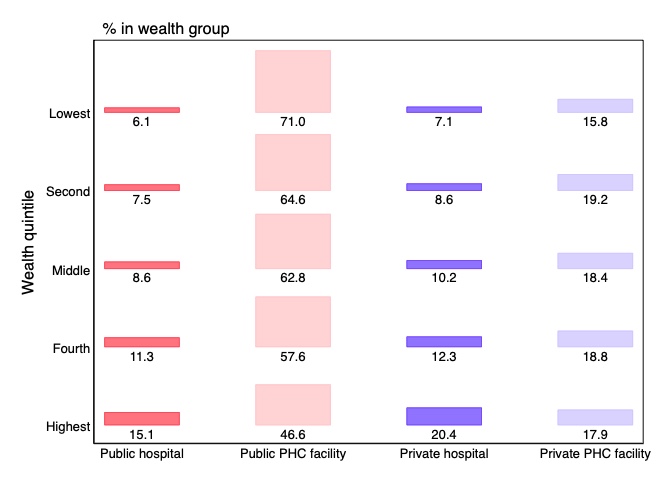


(C) Distribution of the highest education level of children’s parents by facility type


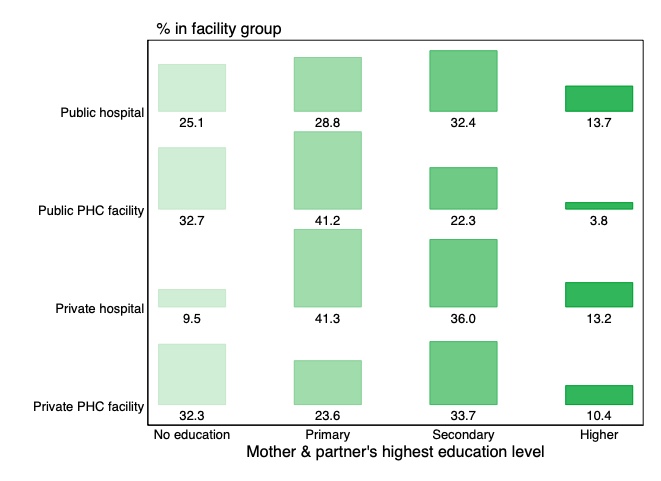


(D) Distribution of wealth quintile by facility type


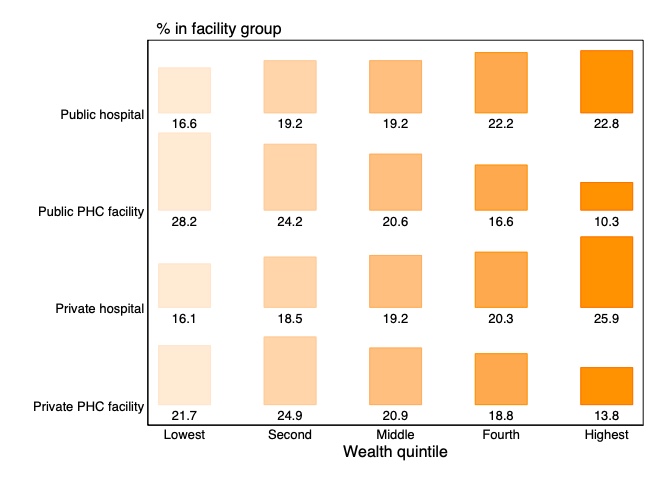

Supplement: Supplementary file 1 — Additional file 1: Table S1. Percentages of febrile children under five who sought care and percentages of care-seekers who received blood tests by country. Table S2. Regional differences in care-seeking behaviors and receipt of blood tests by country. Table S3. Regression results on determinants of care-seeking behaviors and receipt of blood tests for malaria in children. Table S4. Regression results on determinants of blood tests for malaria in children (Stratified analysis). Table S5. Regression results on determinants of care-seeking behaviors and receipt of blood tests for malaria in children (sensitive analyses). Table S6. Predicted probability of receiving blood tests with 95% CIs by mother & partner’s highest education level across facility types. Table S7. Predicted probability of receiving blood tests with 95% CIs by wealth quintile across facility types. Fig. S1. Algorithm for uncomplicated Plasmodium falciparum malaria diagnosis and treatment. Note: This figure was generated by the authors according to the WHO 2021 Guidelines for malaria. a Danger signs for severe falciparum malaria can be found in WHO 2021 Guidelines for malaria[5].b For the treatment after admission, please see World Health Organization, Universal access to malaria diagnostic testing – An operational manual, 2013.c Pre-referral treatment as recommended by WHO 2021 Guidelines for malaria[5]. Fig. S2. Flow diagram of samples for each treatment cascade. Note: All numbers are unweighted. Fig. S3. Crosstabs of facility type and children’s SES. Distribution of facility type by the highest education level of children’s parents. (B) Distribution of facility type by wealth quintile. (C) Distribution of the highest education level of children’s parents by facility type. (D) Distribution of wealth quintile by facility type. [file 40249_2023_1075_MOESM1_ESM.docx]
